# The effects of high-intensity interval training on NLRP3 inflammasome and monocyte chemokine receptors in individuals with obesity

**DOI:** 10.1371/journal.pone.0343214

**Published:** 2026-02-23

**Authors:** Ana Luíza Pereira Assunção Silveira, Daniela Alves de Abreu, Amanda de Lima Santos Musto, Luiz Henrique da Silva Nali, Jônatas Bussador do Amaral, André Luis Lacerda Bachi, Lucas Melo Neves, Saulo Gil, Fernando Mateus Santos, Jefferson Russo Victor, Marina Tiemi Shio, Carolina Nunes França

**Affiliations:** 1 Post Graduation Program in Health Sciences, Santo Amaro University, São Paulo, Brazil; 2 ENT Research Lab. Department of Otorhinolaryngology –Head and Neck Surgery, Federal University of Sao Paulo, São Paulo, Brazil; 3 Division of Dermatology, University of Sao Paulo, Medical School, Sao Paulo, Brazil; University of the Pacific, UNITED STATES OF AMERICA

## Abstract

Background/ Objectives: Obesity is a chronic disease that promotes increased cytokine production mediated by activating a complex of intracellular proteins known as the inflammasome, mainly NLRP3. The modulation of the NLRP3 inflammasome and monocyte chemokine receptors induced by high-intensity interval training (HIIT) in individuals with obesity is still poorly understood. This training is characterized by a period of high-intensity stimulation followed by a recovery period. The main objective of the current study was to investigate possible modulation of the NLRP3 inflammasome and monocyte chemokine receptors in individuals with obesity following HIIT. Subjects/Methods: In this randomized trial, we included individuals with obesity (n = 109), who have not exercised in the last six months, of both sexes, aged between 18 and 60 years. Interventions: Participants were divided into a trained group, which performed three weekly HIIT sessions over eight weeks, and an untrained group (control). The NLRP3 inflammasome and its components, as well as monocyte chemokine receptors expression were analyzed before and after training period by real-time PCR. Results: Whereas NLRP3 expression were unchanged (p = 0.08), after HIIT there was a decrease in Caspase-1 (CASP-1) expression in contrast to the increase of ASC expression as compared to baseline values (p = 0.04 and p < 0.0001, respectively). Additionally, lower IL-6 expression (p < 0.0001), with no differences in IL-1 β and IL-18 expressions were found after HIIT. Besides, HIIT led to an increase in the expression of CCR2 and CCR5 and a decrease in CX3CR1 expression (p < 0.0001, p = 0.001 and p = 0.007, respectively) compared to baseline. Differentially expressed genes interaction analysis [CASP-1, ASC (PYCARD), IL-6, CCR2, CXC3CR1 and CCR5] revealed interaction with NLRP3 nflammasome genes. Conclusions: Eight weeks of HIIT can modulate some components involved in the NLRP3 inflammasome, as well as monocyte chemokine receptors, in individuals with obesity, suggesting an improvement in the control of inflammatory status. **Trial registration**: Brazilian Registry of Clinical Trials – ReBEC number RBR-8vfxfqd).

## Introduction

Obesity is a chronic and multifactorial disease with environmental, biological, psychosocial and socioeconomic factors [1]. It is defined by the World Health Organization (WHO) as an excess of body fat that accumulates in adipose tissue, where on average 40% of macrophages are found, which is directly related to the degree of tissue inflammation [2].

There is a strong link between obesity and systemic inflammation, leading to disturbances in carbohydrate and lipid metabolism and diseases such as type 2 diabetes and atherosclerosis [3–5]. The inflammatory process increases the production of cytokines and the activation of a complex of intracellular proteins called inflammasomes, of which NLRP3 has been the most studied in the metabolic context [6,7]. Its activation occurs by multiple stimuli and causes a cascade of inflammatory processes that can lead to systemic inflammation [8]. The NLRP3 complex is associated with activation of the cytokines, such as interleukin (IL)-1β-mediated inflammatory pathway, which also includes IL-6, the latter playing a causative role in cardiovascular disease [9].

Dysregulation of NLRP3 is associated with the development of several diseases, including obesity. The metabolites of obesity activate the inflammasome, and activation of the inflammasome in turn influences the consequences of obesity [10,11]. Obesity-associated warning signals such as ceramide and palmitate (PA) can activate the NLRP3 inflammasome through the generation of reactive oxygen species, defective mitophagy and a reduction in protein kinase activity. This obesity-induced inflammasome activation in adipose tissue, pancreas and liver promotes chronic inflammation and is associated with metabolic disorders [11].

Chemokines are proteins that belong to a large family of cytokines and, together with their receptors as CCR2, CCR5 and CX3CR1, among others, can control the presence and migration of most cells of the immune system, such as monocytes [12]. Signaling through chemokine receptors plays an important role in the development of adipose tissue inflammation and the subsequent metabolic disturbances in obesity. However, the dual role of chemokines and their receptors in the etiology of obesity-associated inflammation and in energy metabolism has not been well characterized [13,14].

Exercise training is widely recognized for its immunomodulatory benefits, including the modulation of pro- and anti-inflammatory cytokine secretion [15–17]. For instance, a study demonstrated that a 12-week aerobic exercise program significantly reduced inducible Tumor Necrosis Factor (TNF) synthesis in young, healthy, sedentary adults [18]. Similarly, other studies have shown that exercise training provides immunomodulatory effects in individuals with low-grade systemic inflammation, such as those with type 2 diabetes mellitus or chronic heart failure [19,20]. While traditional aerobic training has been shown to provide immunomodulatory benefits, alternative approaches such as high-intensity interval training have gained attention for their potential to achieve similar or even enhanced outcomes [21].

High-intensity interval training is characterized by repeated bouts of high-intensity exercise interspersed with short recovery periods, typically performed at intensities near maximal effort. This method has been proposed as an effective alternative to traditional training due to its time-efficient nature [22]. Moreover, previous studies have indicated that high-intensity interval training may positively influence circulating levels of TNF-α, leptin, and adiponectin, suggesting its potential as an effective and time-efficient intervention for managing low-grade inflammation in individuals with metabolic disorders [21].

Physical exercise (PE) may also have an anti-inflammatory effect on individuals with obesity by modulating not only the activity of the NLRP3 inflammasome but also the phenotype of monocytes and their chemokine receptors. During the inflammatory process, different subtypes of monocytes are recruited from the bone marrow and extramedullary organs, and PE can alter the expression of these receptors. This effect may be particularly important in the context of obesity, where chronic, low-grade inflammation is prevalent and contributes to the development of insulin resistance and other metabolic complications. The modulation of monocytes and their chemokines by PE suggests that this intervention may be an effective strategy to reduce inflammation and improve metabolic health in people with obesity, offering a non-pharmacological approach to treating these conditions [23–25].

There is still a gap regarding the modulations of chemokine receptors and NLRP3 inflammasome by high-intensity interval training (HIIT) in individuals with obesity undergoing HIIT, compared to untrained individuals, since it was reported that several genes may regulate NLRP3 inflammasome and may also be activated by alternative pathways, as previously described [26]. This training is characterized by a period of high-intensity stimulation followed by a recovery period. Hence, understanding the impact of HIIT on NLRP3 gene expression in individuals with obesity may not only help us to elucidate the dynamics of this inflammasome expression, as well as other genes, but also may contribute to improving our knowledge regarding the benefit of this non-pharmacological intervention that provides good adherence and benefits the population with obesity.

In this sense, the aim of this study was to investigate a possible modulation of the NLRP3 inflammasome, including its main components, and monocyte chemokine receptors by HIIT in individuals with obesity.

## Materials and methods

### Study design and sample size calculation

**Fig 1:**
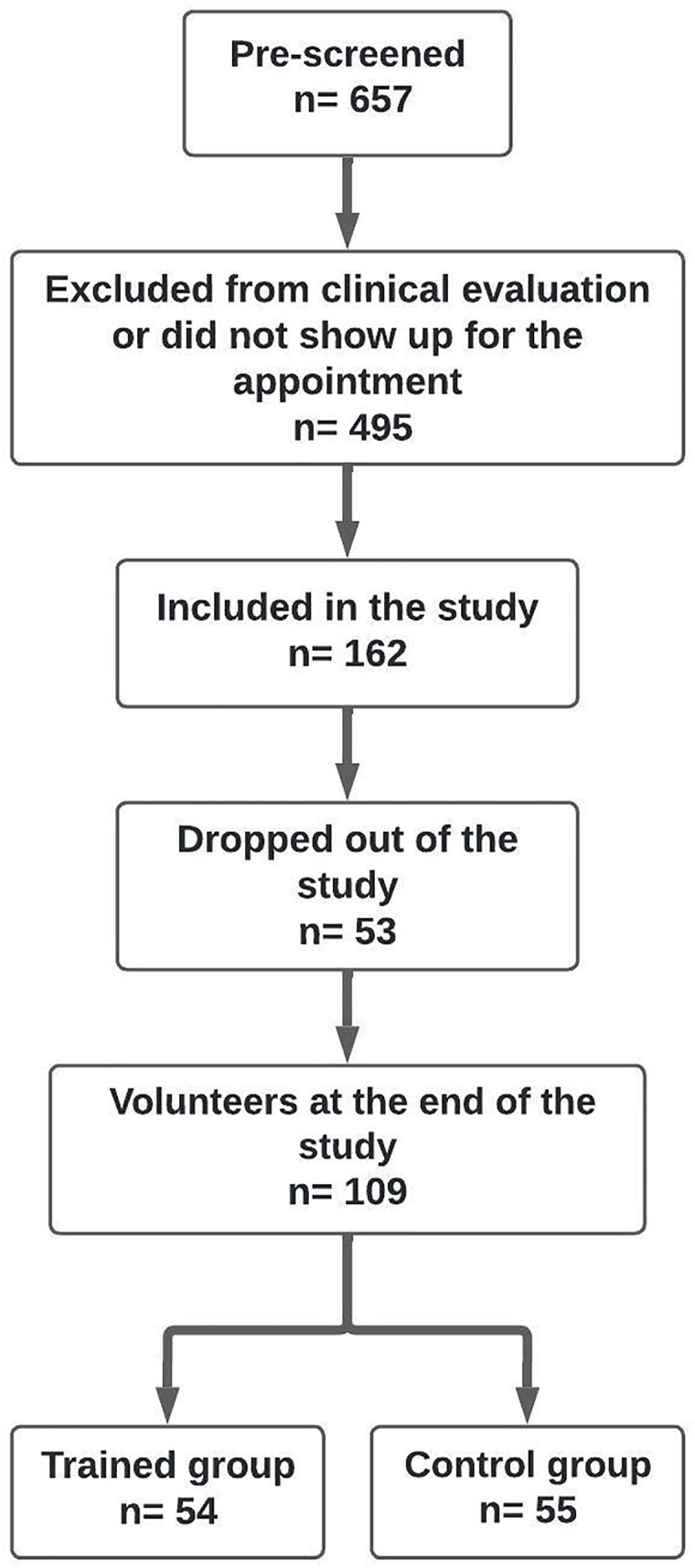
Flow chart of the study.

Prospective, controlled and randomized study, with experimental and control groups. The effects of eight weeks of HIIT were evaluated. The groups consisted of adults with obesity [Body Mass Index (BMI) between 30 and 40 kg/m2] who did not exercise in the last six months of both sexes and aged between 18 to 60 years old (Fig 1, Supporting Material 1 Material 1 and Supporting Table 1).

**Table 1:** General characteristics of the sample at the baseline visit.

	Trained Group	Control Group	p
N	54	55	
F/M	46/8	44/11	0.48^*^
Age, years (mean±SD)	45 (10)	44 (11)	0.81^#^
Weight, Kg (mean±SD)	88.61 (15.43)	90.56 (13.29)	0.51^#^
Height, m (mean±SD)	1.62 (0.09)	1.61 (0.07)	0.95^#^
BMI, Kg/m^2^ (mean±SD)^$^	34.14 (3.58)	34.34 (3.85)	0.79^#^
Diabetes, n (%)	10 (19)	11 (20)	0.95*
Hipertension, n (%)	15 (28)	11 (20)	0.48*

F/M: Female/Male; SD: Standard Deviation; BMI: Body Mass Index.

*Pearson Qui-Square Test; ^#^Unpaired T-test.

Only volunteers were included in the study after they had been clinically assessed by the study medical team and certified that they were able to engage in the exercise training. Participants were recruited (25^th^ October 2022 to 10^th^ June 2024) from both the Community Extensive Sports Activities Program (PAEC) and Nutrition Clinic of the Santo Amaro University (UNISA) database.

The sample size was calculated based on previous studies that have considered around 30 individuals per group. However, we have focused on an effect size of 0.55 considering the interval confidence value of 90% and significance alfa risk of 5% and 80% of power to detect the significance, the results revealed around 84 volunteers, however we have considered a rate of 10% loss of individuals during the follow up. In this sense, we have estimated a total of 92 individuals. Fortunately, we have concluded the follow up with a total of 109 participants, the analysis was performed on the software G*Power Program, version 3.1.9.4 [27]. The randomization of groups was carried out using the jerrydallal platform. Participants were randomly allocated in a 1:1 ratio into one of the following experimental groups: (a) Trained group, or (b) Control group.

### Physical exercise protocol

**Fig 2:**
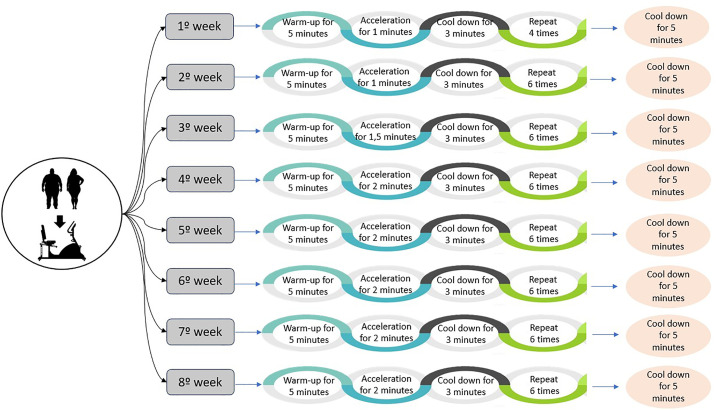
Protocol of training.

The physical exercise program was carried out for eight weeks, three times a week, on a cycle ergometer (V3 Vertical Exercise Bike, Movement, Brazil) with a protocol recommending a gradual increase in the stimulation time and repetitions of the training used (Fig 2, Supporting Material 1 and Supplementary S1 Fig). At the beginning and end of each exercise session, a five-minute warm-up was performed and then the volunteer returned to rest. Regarding the training itself, to adapt to the protocol, in the first week the volunteer was subjected to 4 1-minute stimuli at high intensity (80 to 100% maximum heart rate - HRmax), followed by 3 minutes of recovery (50 to 70% HRmax). In the second week, the volunteer was subjected to 6 1-minute stimuli at high intensity (80 to 100% HRmax), followed by 3 minutes of recovery (50 to 70% HRmax). In the third week, the volunteer was subjected to 6 stimuli of 1 minute and thirty seconds at high intensity (80 to 100% HRmax), followed by 3 minutes of recovery (50 to 70% HRmax). From the fourth week and until the end of the training period, the volunteer was subjected to 6 stimuli of 2 minutes at high intensity (80 to 100% HRmax), followed by 3 minutes of recovery (50 to 70% HRmax) [28,29].

The intensity of the stimulus was monitored using a heart rate monitor with Bluetooth transmission (model H7, Polar, Finland), which transmitted the data to the Polar Team program (Polar Team, Finland) installed on a tablet or smartphone or recorded on the Polar watch. The data was based on the predicted maximum heart rate accompanied by the subjective perception of effort (Borg scale), confirming that the target range of physical exercise recommended for each subject was achieved in each training session (Supplementary S1 Fig).

### Lipid profile

Circulating serum levels of total cholesterol and fractions (low-density lipoprotein cholesterol – LDL-C and high-density lipoprotein cholesterol – HDL-C), triglycerides, albumin, total protein, uric acid, glucose (LabTest diagnostica, Lagoa Santa, Brazil) and peroxide (Bioassay Systems-Hayward, United States) were determined with commercially available kits. The values obtained were analysed using a Multiskan Sky Spectrophotometer microplate reader (Thermo Fisher Scientific—Vienna, Austria). LDL-C values were estimated according to the Friedewald formula [30].

### Body composition analysis

Body composition was assessed at the beginning and after the recommended physical training period using a densitometer using dual-energy X-ray absorption (DEXA). Total body image will be acquired using GE Healthcare Lunar equipment (iDXA Madison, WI, USA). Individuals were positioned in the equipment wearing only light clothing. Lean and fat tissue mass measurements will be taken (percentage of total fat, by region, android fat and gynoid fat). Body composition analysis will be carried out in partnership with a private laboratory.

### Collection of mononuclear cells from peripheral blood

Peripheral blood samples were collected fasting in EDTA-containing tubes to avoid clotting. Approximately 15 mL of blood was collected at baseline and after eight weeks of training. The blood sampling in the trained volunteer group occurred 24–48 hours after the last HIIT session.

The blood was centrifuged for 10 minutes at 800 g and 23⁰C to remove excess plasma content, diluted with phosphate-buffered saline (PBS), ratio 1:1, and then transferred to a 15 mL Falcon tubes containing Ficoll-Hypaque (Ficoll Paque Plus, GE Healthcare Bio-Sciences AB, Uppsala, Sweden. After centrifugation at 800 g for 20 minutes at 23ºC, the peripheral blood mononuclear cells (PBMC) were harvested, transferred to a new tube, washed in PBS, submitted to a new centrifugation round (10 minutes at 800 g and 23⁰C) and after discarding the supernatant, 3 ml of ammonium chloride was added for two minutes at room temperature to lyse the remaining red blood cells. The cells were washed again with PBS, submitted to a new centrifugation round (10 minutes at 800 g and 23⁰C), and the supernatant was discarded. One milliliter (1 mL) of PBS was added to resuspend the pellet, which was transferred to microtubes, and after centrifugation (10 minutes at 800 g and 23⁰C), the supernatant was discarded, and the dry pellet was stored in a freezer at −80ºC.

### RNA isolation and reverse transcription

To study the expression of NLRP3 inflammasome (and associated components) and monocyte chemokine receptors, PBMC were subjected to RNA extraction with Trizol as described by Rangel et al. (2024) [31]. Samples were treated with DNAse (Turbo DNA-free kit, Thermofisher) to remove contaminating genomic DNA. The Nanodrop 2000 device (Applied Biosystems) was used to assess the concentration and quality of RNA present in each sample. The Superscript II Reverse Transcriptase Kit (Thermofisher) was used to recover the cDNA (reverse transcription). We used GAPDH as a control gene.

### Gene expression

As described by da Silva (2023) [32], each sample contained 2X TaqMan Gene Expression Master Mix (Thermofisher) – 10 μL, DNAse and RNAse free water, 1 μL cDNA and 1 μL 20X TaqMan Gene Expression Assay for a total of 20 μl per reaction. Experiments were performed in duplicates and reactions were analysed using the StepOnePlusTM Real-Time PCR System.

The primers used are listed in Supporting Table 2.

**Table 2:** Biochemical parameters of the participants.

Parameter	Trained Group		Intra groups p	Control Group		Intra grupos p	Between groups p
	T0	T1		T0	T1		
Total Cholesterol^*^	306 (225-365)	253 (174-317)	0.309	263 (192-333)	229 (191-278)	0.189	**0.005**
Triglycerides^*^	157 (132-193)	151 (129-169)	**0.009**	153 (129-211)	145 (125-214)	0.404	0.190
LDL-C^$^	223 (162-287)	190 (117-251)	0.666	182 (112-259)	157 (115-207)	0.227	**0.031**
HDL-C^*^	41 (38-43)	38 (36-44)	0.088	41 (37-49)	42 (38-65)	0.248	0.680
Non-HDL-C^*^	261 (184-320)	214 (137-281)	0.351	207 (139-281)	191 (146-225)	0.337	**0.017**
Glucose^*^	107 (89-127)	92 (86-115)	0.280	99 (88-118)	95 (86-127)	0.157	0.153
Uric Acid^*^	5.3 (4.8-6.3)	4.8 (4.3-5.8)	**0.040**	5.1 (4.7-6.2)	5.0 (4.2-5.8)	0.109	0.451
Albumin^#^	6.0 (5.6-6.5)	4.0 (3.7-4.4)	**0.016**	5.9 (5.6-6.3)	4.1 (3.8-4.4)	**0.005**	0.199
Total Proteins^&^	6.4 (5.9-7.3)	4.2 (3.9-4.9)	**0.044**	6.6 (5.8-7.2)	4.5 (4.0-5.0)	**0.002**	0.664

* mg/dL; ^#^g/L; ^&^g/dL; ^$^Calculation using the Friedwald formula. Data represented as medians (interquartile range). Wilcoxon test, intra-group comparison (initial time – T0 and final time – T1 in each group). Kruskal-Wallis test, comparisons between groups.

The level of expression of each gene was determined by 2-ΔΔCt method considering GAPDH gene as the housekeeping gene to normalize the expression among the samples. The results were expressed as fold change.

### Gene network analysis

Differentially expressed genes (p < 0.05) were uploaded in IntAct molecular interaction analysis data base [33], to determine possible genic pathways among the differentially expressed genes. For control, we have only included genes with direct interaction and association, also we have included genes which the interaction method included: ubiquitin reconstruction, anti tag and anti coip methods and MI confidence interval over 0.5 were considered.

### Statistical analysis

For the analysis of the descriptive measures and the statistical tests, the program SPSS version 23.0 is used, whereby parametric and non-parametric tests are used depending on the type of variable.

The Kolmogorov-Smirnov test is used to test the normality of the variables. Descriptive data will be expressed as percentage, mean ± standard deviation or median and interquartile range where appropriate. Comparisons between groups (trained and control) were made using the unpaired t-test or Mann-Whitney test. For comparisons between visits in the trained group (first visit, before the start of the training protocol and at the end of the eight-week training period), the paired t-test and Wilcoxon test were used. A significance level was set at p < 0.05.

### Ethical aspects

Participants read and signed the free and informed written consent form (TCLE). The study was approved by the Research Ethics Committee of the University of Santo Amaro (number 5.707.974), registered as a clinical trial before its start (Brazilian Registry of Clinical Trials – ReBEC number RBR-8vfxfqd) and followed the principles described in the Declaration of Helsinki.

## Results

Fig 1 shows the detailed flow chart of the study. Patients (n = 657) were pre-selected for clinical evaluation. Of these, 54 participants were included in the trained group and 55 in the control group. In the control group, participants were instructed not to exercise for eight weeks and samples were also taken on two visits.

Table 1 shows the general data of the participants, with no differences between the control and trained groups on the first visit (baseline values).

### Lipid profile

The biochemical parameters are listed in Table 2. After eight weeks of HIIT, there was a reduction in triglycerides (p = 0.009), with no differences in total cholesterol, LDL-C, HDL-C, non-HDL-C and glucose (p = 0.309, p = 0.666, p = 0.088, p = 0.351 and p = 0.280, respectively). After comparing the two groups, there were differences in total cholesterol, LDL-C and non-HDL-C (p = 0.005, p = 0.031 and p = 0.017, respectively) with no differences in triglycerides, HDL-C and glucose (p = 0.190, p = 0.680 and p = 0.153, respectively).

In addition, after eight weeks, there was a reduction in albumin and total proteins in both the trained and control groups (p = 0.016, p = 0.044, p = 0.005 and p = 0.002, respectively. Wilcoxon test) and a reduction in uric acid in the trained group (p = 0.040) with no differences in the control group (p = 0.109); there were no differences between the groups (p = 0.199, p = 0.664 and p = 0.451. Kruskal Wallis test).

### Density Measurement (DEXA)

Table 3 shows the results of the bone density measurement (DEXA) obtained in the trained and control groups. There were no differences in the percentage of adipose tissue, fat mass, lean mass and total mass when comparisons were made between T0 and T1 for each group (Wilcoxon test); there were also no differences after comparisons between the groups (Kruskal Wallis test).

**Table 3:** Analysis by Bone Densitometry – DEXA, intra-group comparisons (initial time – T0 and final time – T1 for each group) and between groups (Trained and Control).

Parameter	Trained Group		Intra grous p	Control Group		Intra grous p	Between groups p
	T0	T1		T0	T1		
Percentage of tissue fat (%)	48 (43-52)	47 (41-52)	0.184	47 (40-52)	45 (38-55)	0.893	0.670
Fat mass (Kg)	40 (32-49)	35 (29-44)	0.709	39 (32-45)	35 (27-45)	0.500	0.210
Lean mass (Kg)	43 (37-49)	43 (37-50)	0.062	42 (37-51)	41 (37-47)	0.893	0.912
Total mass (Kg)	87 (74-97)	83 (73-95)	0.444	82 (76-95)	79 (75-87)	0.680	0.410

Data represent medians (interquartile range). Wilcoxon test, intra-group comparison (initial time – T0 and final time – T1 in each group). Kruskal-Wallis test, comparisons between groups.

### Gene expressions

The analyzes considered the 2 − ΔΔCt method, which calculates the value of the fold change in gene transcription levels. A value of 1 corresponds to no change in gene expression, while values above 1 indicate an increase and below 1 a decrease in gene expression.

### NLRP3, Caspase-1 (CASP-1), ASC, IL-1b, IL-6 and IL-18 expressions

**Fig 3:**
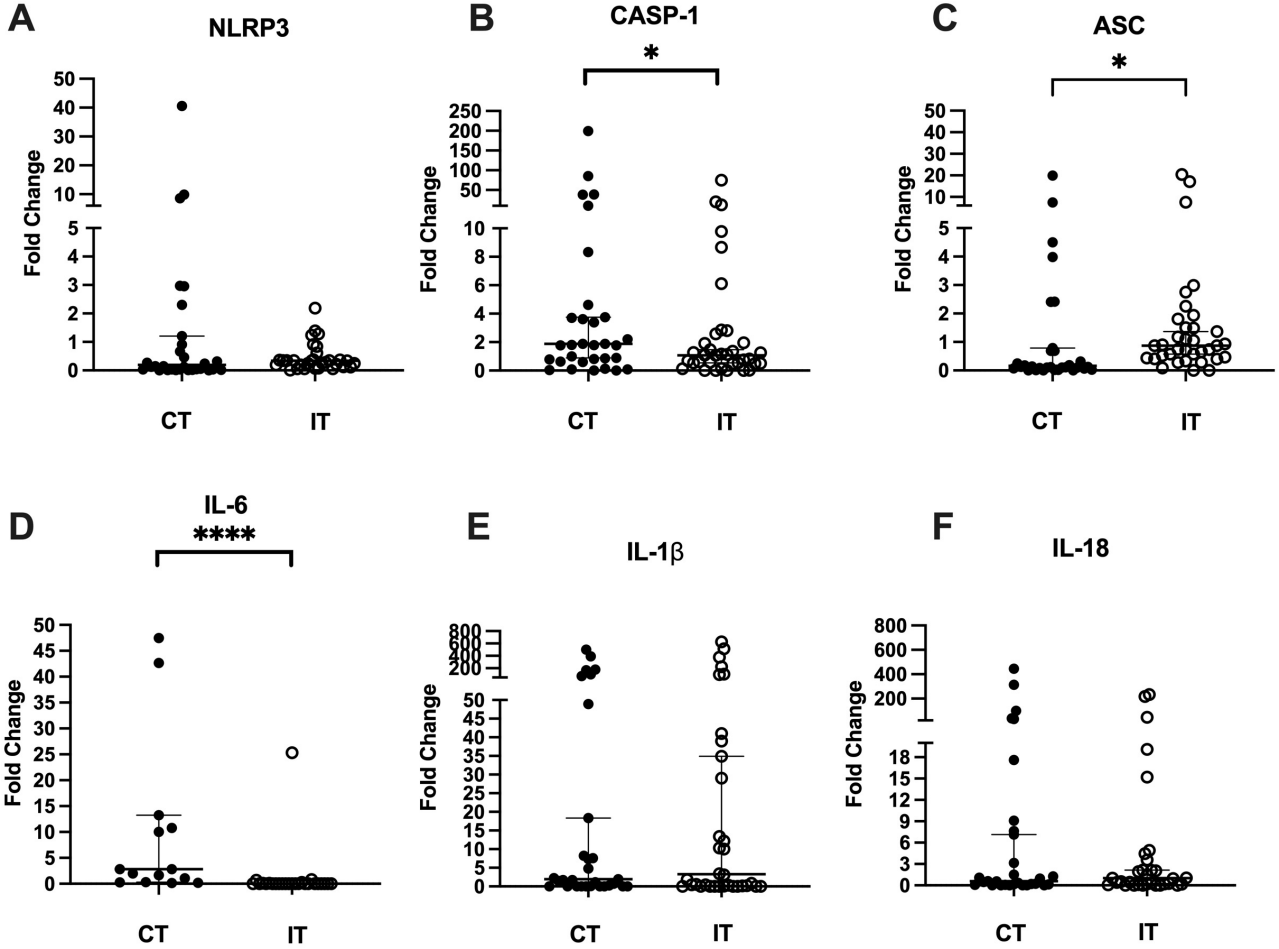
Expression of the genes A) NLRP3, B) CASP-1, C) ASC, D) IL-6, E) IL-1β, and F) IL-18. There was a reduction in CASP-1 (p = 0.04) and an increase in ASC (p < 0.0001) in the Trained Group (IT). Mann-Whitney test. Among the interleukins tested, there was a reduction in IL-6 (p < 0.0001) in the Trained Group, without differences to IL-1β and IL-18. Mann-Whitney test.

After a comparison between the trained and control groups, there were no differences for NLRP3 expression, but a reduction in CASP-1 expression (p = 0.04) and an increase in ASC after eight weeks of training (p < 0.0001), although in this case it kept lower than 1. Among the interleukins tested, there was a decrease in IL-6 (p < 0.0001) and no differences in IL-1β and IL-18 in the trained group (Fig 3).

### CCR2, CCR5 and CX3CR1 expressions

**Fig 4:**
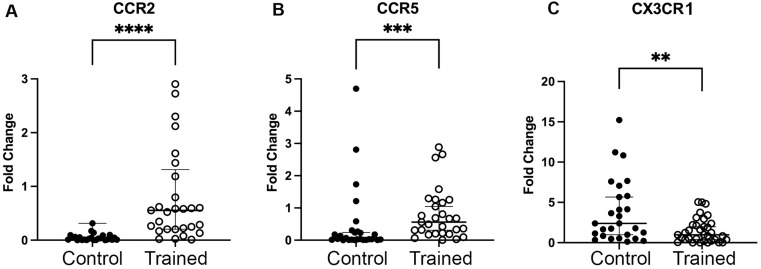
Expression of the genes A) CCR2, B) CCR5, and C) CX3CR1. There was an increase in the expressions of CCR2 and CCR5, and a reduction in CX3CR1 in the Trained Group (p < 0.0001, p = 0.001 and p = 0.007, respectively). Mann-Whitney test.

Analysis of monocyte chemokine receptors showed an increase in the expression of CCR2 and CCR5 and a decrease in CX3CR1 in the trained group (p < 0.0001, p = 0.001 and p = 0.007, respectively. Fig 4), without differences in the control group. There were no differences after comparisons between the two groups.

### Genic pathway analysis

Differentially expressed genes interaction analysis (CASP-1, ASC (PYCARD), IL-6, CCR2, CXC3CR1 and CCR5) revealed interaction with several genes (Supplementary S2 Fig), some of them were among the NLRP3 inflammasome genes, and interestingly other genes may also show direct interaction and/or association with differentially expressed genes. Genic pathway of each differentially expressed genes can be found in the Supplementary S2 Fig. Each gene/protein function (according to uniProt - https://www.uniprot.org/) are shown on Supporting Table 3.

## Discussion

The results of the present study, which evaluated the impact of HIIT on the modulation of genes related to not only the NLRP3 inflammasome, and its components, which including inflammatory cytokines, but also monocyte chemokine receptors, showed, for the first time, a significant reduction in the expression of the CASP-1 and IL-6 genes, in contrast to an increase in the ASC gene expression in the trained group. This pattern suggests a selective modulation of the inflammasome signaling axis, with greater impact on adaptor and effector components rather than on NLRP3 transcription itself, which is consistent with previous observations that NLRP3 activity is frequently regulated post-transcriptionally [26].

The results concerning CASP-1 and IL-6 both agree and reinforce the literature findings that describe HIIT as a promising intervention for modulating inflammatory processes, particularly those related to obesity. Previous studies suggest that the performance of aerobic exercise three times a week for 6 weeks may reduce inflammasome activation and inflammatory cytokine release, thereby improving insulin sensitivity and other metabolic markers in individuals with obesity [34]. These effects may be mediated by the reduction of cellular stressors, such as reactive oxygen species (ROS), which play a critical role in NLRP3 inflammasome activation [26].

In addition, studies have shown that PE, particularly at high intensity, can attenuate the expression of caspases, proteins involved in cell apoptosis and inflammatory processes mediated by the NLRP3 [35]. For instance, CASP-1, which is activated by the NLRP3 inflammasome, plays a central role in the maturation of the pro-inflammatory cytokines IL-1β and IL-18, thus its reduction may lead to a decrease in systemic inflammation [36,37]. Notably, this reduction in CASP-1 expression may occur even in the absence of changes in NLRP3 gene expression, as the activation of the inflammasome is regulated by cellular stress signals such as ROS [26]. Since HIIT can reduce ROS production and other intracellular stressors, it may suppress NLRP3 inflammasome activity without altering its transcriptional levels [34]. This finding is consistent with studies in metabolic disease showing discrepancy between NLRP3 mRNA levels and CASP-1 activity, indicating that exercise may preferentially act on signal 2 (activation) rather than signal 1 (priming) [26,34,36].

Butts and colleagues (2018) [9] investigated the effects of three months of aerobic training on the expression of ASC, IL-18 and iNOS genes in people with heart failure. There was an increase in ASC expression in the trained group compared to the control group after three and six months of training, and lower expression in the trained group when baseline was compared to three months, and six months of training. In another study, Barrón-Cabrera and coworkers (2020) [38] investigated the effects of PE with moderate intensity for four months in conjunction with diet on the regulation of ASC in individuals with obesity and found a reduction in ASC expression in the group that received the diet + exercise association compared to the group that was placed on diet alone. In contrast, our study found an increase in ASC expression in the trained group after eight weeks, although the fold change kept lower than 1.0 after the training, which points to a low expression of the gene. Some considerations need to be made when evaluating and comparing our results with Butts’ study. In Butts’ work, people with heart failure were examined; in our study, it is participants with obesity. In addition, the subjects in Butts’ study were given instructions for a moderate-intensity exercise protocol to perform at home and were monitored remotely through regular telephone contact. In relation to the work of Barrón-Cabrera, [38] there was an assessment of diet in relation to exercise, and the proposed training was of moderate intensity, two parameters that differentiate the above study and our work. Moreover, ASC expression has been shown to be sensitive to both metabolic status and inflammatory milieu, and it is possible that the early increase observed here represents a transient adaptive response before a later downregulation, as suggested by longer intervention studies [37,38]. There are still few studies investigating the effects of exercise specifically on the expression of ASC mRNA, and the apparently contradictory results confirm what has already been shown in the previous findings, namely that the target population and the training time are crucial for defining the changes in the markers studied.

In the present study, no significant differences were found in the levels of IL-1β and IL-18, suggesting that although the CASP-1 gene was modulated by HIIT, this modulation may not have been sufficient to cause significant changes in the release of these inflammatory cytokines. This is consistent with the hypothesis that different intensities of exercise can elicit different responses. While HIIT has shown efficacy in reducing inflammatory markers in some studies, intensity and duration may be critical factors in achieving more robust effects. In addition, IL-1β and IL-18 are tightly regulated at the post-transcriptional level and require active inflammasome assembly for secretion, which may explain the absence of significant changes despite reduced CASP-1 expression [26,36]. Similar dissociations between CASP-1 modulation and IL-1β levels have been reported in exercise interventions in metabolic disorders [34].

The results of the current study concerning the monocyte chemokine receptors CCR2, CCR5 and CX3CR1 showed an increase in the expression of CCR2 and CCR5 and reduction in CX3CR1 expression after HIIT in individuals with obesity. In accordance with our results, Nowak et al (2023) [39] found increased expressions of CCR2 and CCR5 after aerobic exercise without differences concerning anaerobic exercise, among younger individuals. The increase in CCR2 and CCR5 may reflect enhanced monocyte mobilization and trafficking, a physiological response to exercise-induced tissue remodeling and repair [39,40].

In another study, Barry et al (2017) [40] compared the effects of HIIT and moderate-intensity continuous training (MICT) on the chemokine receptors of individuals with obesity and they found that 2 weeks of MICT reduced CCR2 and HIIT was able to increase CCR5 on monocytes, showing that the type of exercise training is crucial in the modulation of the chemokine receptors. This supports the concept that HIIT and MICT elicit distinct immunological adaptations, possibly due to differences in sympathetic activation, myokine release, and shear stress [25,40].

Another studies, on the other hand, suggest that modulation of these receptors is more likely to be related to long-term adaptations, such as reducing low-grade inflammation and improving metabolic health in response to chronic exercise [41]. Decreasing levels of inflammation, often observed with prolonged exercise programs, may be associated with more gradual changes in chemokine receptor expression and immune cell migration. The reduction in CX3CR1 observed in our study is consistent with a potential shift away from a more pro-inflammatory and endothelial-adherent monocyte phenotype, as CX3CR11-high monocytes have been associated with vascular inflammation and atherogenesis [14,41].

Additionally, the genic pathway analysis revealed several genes that might be regulated by the expression of the NLRP3 inflammasome genes. These genes are associated with immune response, inflammation, apoptosis, activation, and structural proteins (cytoskeleton, membrane and endosome formation) (Supplementary S2 Fig). Although NLRP3 gene expression itself was unchanged, downstream components like CASP-1 and ASC are related independently. This is in line with studies showing that inflammasome signaling can be modulated through non-canonical pathways and cross-talk with other pattern recognition receptors, which may be particularly relevant in the context of exercise-induced immune adaptation [11,26].

In fact, ASC (PYCARD-1), may interact with other inflammassome proteins such as NLRP1 and NLRC4, thus the upregulated genes might indicate that volunteers after the training protocol could be in a pro-inflammatory status, although IL-6 expression may reveal that a control mechanism could also be elicited. Of note, the upregulation of ASC (PYCARD-1), could also reveal a positive scenario, since it interacts with CHUK, SYK, and IKBKB, which are genes that plays several roles: e.g., CHUK acts preventing pyroptosis through inactivating NF-κB signaling, SYK play several physiological roles such as activation of lymphocytes and macrophages, platelets activation and vascular development and hematopoiesis, also in animal model SYK is required for survival and B cell development. Finally, IKBKB plays a crucial role in innate response against bacterial and viral infections and DNA damage or other cellular stresses, importantly, mutations in the IKBKB genes induce immunodeficiency. In this sense, the training protocol reflects an upregulation of ASC (PYCARD1) which interact with crucial genes that may be regulated by this expression which could both improve immune response and physiological conditions after HIIT protocol. Therefore, the observed ASC modulation should be interpreted with caution and within the context of a broader immune regulatory network, rather than as an isolated pro-inflammatory signal [26,37,38].

Although it was not the study’s main objective, the evaluation of circulating metabolic markers, particularly glycemia and lipid/protein profiles, was carried out to verify the systemic HIIT effect since it has been reported that this training type can significantly alter these markers. Corroborating our findings, a recent systematic review and network meta-analysis evidenced that HIIT was the most effective training type to improve circulating triglycerides levels in adults with overweight and obesity [42]. Beyond these observations, the literature highlights that increased triglyceride levels are associated with elevations of uric acid in individuals with obesity [43], thus the reduction of circulating triglycerides levels could favor the decrease in uric acid levels evidenced in the same volunteer group. Moreover, the conjunct significant reduction of systemic levels of albumin and total protein was expected, since albumin is the most abundant circulating protein, and can indicate that the HIIT was able to modulate nutritional aspects in trained volunteers. According to the literature, serum albumin levels not only can increase with higher protein intake but also are considered one of the pillars of nutritional status [44], as well as has been correlated with a systemic pro-inflammatory status [45]. In the context of obesity, reductions in albumin may also reflect changes in inflammatory burden and hepatic acute phase response rather than true protein malnutrition [45]. Besides, we found significant differences between the volunteer groups regarding some metabolic markers (total cholesterol, LDL-C, and non-HDL-C), in which, unexpectedly, the control group showed reduced levels of these parameters as compared to the trained group. This may be related to uncontrolled dietary modifications, use of lipid-lowering medication, or lifestyle changes in the control group during follow-up, which could not be fully controlled in the study design [42].

Lastly, it is important to cite that even though this study presents some strong aspects, such as (i) the rigorous process involved in the volunteer’s recruitment and selection, (ii) the controlled conduction of HIIT performance, (iii) the expression analysis of NLRP3, including its main components, as well as of monocyte chemokine receptors by RT-PCR, and also (iv) the assessment of genes interaction, some limitations can be mentioned, such as (v) the number of volunteers in each group, which although reached the necessary number to perform this study, do not allow us to affirm some aspects, (vi) the lack of information regarding diet habits, as well as the use of supplements, e.g., vitamins and antioxidant products, by the volunteers, mainly those of control group, which could putatively justify some significant alterations verified particularly in the circulating protein and lipid profile in this group, (vii) the absence of protein-level measurements and functional assays limits the interpretation of gene expression changes in terms of actual inflammasome activity, (viii) predominance of female participants and the age variability within the cohort, which may have influenced immune and inflammasome-related responses to HIIT and should be considered when interpreting the results.

## Conclusions

Our findings showed, for the first time, that 8 weeks of HIIT is a useful intervention in order to modulate some important components involved in the NLRP3 inflammasome, as well as monocyte chemokine receptors, in individuals with obesity, which, in general, can lead to an improvement in the physio-metabolic conditions and immune responses. In addition, this type of PE promoted both good adherence and did not promote lesions in our volunteers, showing security for this population performs it to alter its sedentary condition to a better lifestyle.

## Supporting information

S1 File Study protocol. (DOCX)

S1 Table CONSORT checklist. (DOCX)

S2 Table Genes and respective *primers* of the study. (DOCX)

S3 Table Function of the genes/proteins evaluated by genic pathway interaction. (XLSX)

S1 Fig Physical parameters during the protocol of exercise. A) Recovery Heart Rate, B) Maximum Heart Rate, C) Recovery Rate of Perceived Exertion; D) Maximum Rate of Perceived Exertion.
(JPG)

S2 Fig Genic pathway of the expressed genes. A) CASP-1; B) PYCARD; C) IL-6; D) CCR2; E) CCR5; F) CX3CR1.
(JPEG)
