# Supplementary material for: The effects of high-intensity interval training on NLRP3 inflammasome and monocyte chemokine receptors in individuals with obesity
Source: PLoS One. 2026 Feb 23;21(2):e0343214. doi: 10.1371/journal.pone.0343214 (PMC12928487; doi:10.1371/journal.pone.0343214)
Supplement: S1 File — (DOCX) [file pone.0343214.s001.docx]

**Possible modulation of NLRP3 inflammation by physical exercise**

Santo Amaro University - UNISA

Responsible researcher: Carolina Nunes França

Beneficiary: Ana Luíza Pereira Assunção Silveira

**ABSTRACT:** Obesity is a chronic disease that affects millions of people worldwide. Currently, the prevalence of overweight and obesity affects approximately 1/3 of the world’s population today. It is known that obesity is closely associated with inflammation, which leads to increased production of cytokines mediated by the activation of a complex of intracellular proteins called inflammasomes, among which the most studied in the metabolic context has been NLRP3. Although it is accepted that physical exercise brings benefits associated with obesity, little is known about the modulation of the NLRP3 inflammasome. Thus, the aim of the present study is to evaluate a possible modulation of the NLRP3 inflammasome in subjects with obesity after high-intensity interval training compared to untrained subjects. Sedentary individuals with obesity (approximately 84) from both sexes, aged between 18 and 60 years, will be invited to voluntarily participate in the present study and then separated into two groups: trained (three weekly HIIT sessions, for a period of eight weeks) and untrained group (control). Assessment of body composition will be performed in a densitometer, through the absorption of dual energy X-rays (DEXA); the NLRP3 inflammasome (and associated interleukins: IL-1β, IL-18 and IL-6) will be evaluated by real-time PCR, before the beginning of the training protocol and after eight weeks of training (concerning the control group, there will be a unique evaluation). Interaction analysis of gene pathways of the NLRP3 inflammasome will also be performed.

**INTRODUCTION**

Obesity is a chronic disease with multifactorial causes (biological, environmental, psychosocial, socioeconomic) that has become a public health problem in recent years. It affects millions of people in both developed and developing countries and is a risk factor for the development of cardiovascular disease, chronic kidney disease, hypertension, type 2 diabetes and others. Since the 1980s, the prevalence of overweight (Body Mass Index - BMI between 25.0 and 29.9 kg/m2) and obesity (BMI over 30.0 kg/m2) has doubled worldwide, affecting 1/3 of the world's population.1-4

Many studies have shown the link between obesity and chronic inflammation, with an increase in cytokine production and activation of a complex of intracellular proteins called the inflammasome (including NLRP3, which is investigated in this study), which upon activation by various stimuli (such as high glucose levels, free fatty acids, cholesterol),5,6 leads to the activation of caspase 1, which converts pro-IL-1β and pro-IL-18 into the cytokines IL-1β and IL-18, which trigger the inflammatory process.7-9 Dysregulation of NLRP3 has been linked to the development of several diseases, including obesity.10

Unamuno et al. (2021)11 investigated the expression levels of NLRP3 inflammasome components in the context of obesity. The authors showed, among other things, that the expression of NLRP3 and associated cytokines (IL-1β and IL-18) is increased in the liver of individuals with obesity associated with type II diabetes.

An important study that investigated the blockade of IL-1β by the use of a monoclonal antibody (canakinumab) in patients with a history of myocardial infarction was the CANTOS (Canakinumab Antiinflammatory Thrombosis Outcome Study) trial. The authors showed that treatment with canakinumab every three months reduced the rate of cardiovascular events and IL-6 levels compared to the placebo group, with no changes in cholesterol or blood pressure. This study suggests that reducing inflammation reduces cardiovascular outcomes even without changes in traditional risk factors such as dyslipidemia or arterial hypertension.12-14 The same CANTOS study also showed that lowering IL-6 levels below the median with the use of canakinumab was associated with a reduction in both cardiovascular mortality (-52%) and all-cause mortality (-48%).15 Another study (ASSAIL-MI) examined IL-6 receptor inhibition (with the drug tocilizumab) in patients after acute myocardial infarction with ST-segment elevation. An improvement in the myocardial salvage index and a reduction in the extent of microvascular obstruction were observed using magnetic resonance imaging.16

Akra et al. (2022)17 investigated the gene expression and secretion of proteins related to the NLRP3 inflammasome in the adipose tissue of patients with coronary artery disease undergoing cardiac surgery. The authors showed an increase in IL-18 and IL-6 in the participants' epicardial adipose tissue, although individuals with obesity were not studied.

The benefits of physical activity for the prevention and treatment of obesity are well established in the literature. In the context of the NLRP3 inflammasome, Mejías-Peña et al. (2017)18 showed in older men that the group that performed resistance training for eight weeks showed less activation of the NLRP3 inflammasome compared to the control group. In another study by Zhang et al. (2023)19, the effect of six months of resistance training combined with Yijinjing (an exercise based on traditional Chinese medicine theory) was evaluated in prediabetics and among the variables modulated by the training, there was an inhibition of NLRP3 inflammasome activity in elderly prediabetics. Another study by Abkenar et al. (2019)20 indicated that the modulation of the NLRP3 inflammasome may depend on the intensity and duration of exercise, as the authors found no differences in the expression of NLRP3, IL-1β and IL-18 associated with moderate-intensity acute exercise, but the same acute exercise, when performed at high intensity, promoted the increase in the inflammasome and interleukins. It was also shown that moderate aerobic exercise led to a reduction in NLRP3, IL-1β and IL-18 after 12 weeks.

Armannia et al. (2022)21 compared the effects of high-intensity interval training (HIIT) with moderate-intensity continuous training (MICT) on NLRP3 expression in obese individuals who exercised three times per week for eight weeks. The results showed that both training protocols downregulated NLRP3 expression compared to the control group. However, one of the limitations of the study was the small number of participants (n=12 in each group).

Regarding the benefits of HIIT for obese individuals, in a meta-analysis involving 19 studies, Sabag et al. (2022)22 assessed the effects of HIIT compared to MICT on liver fat in 745 individuals and showed that both exercise groups resulted in a moderate reduction in liver fat compared to a control group; however, the authors suggest that HIIT may be more effective as it requires a lower energy expenditure and is performed in a shorter time. HIIT improves cardiorespiratory fitness in overweight and obese individuals,23 blood glucose control, blood pressure reduction24 and waist circumference reduction.25 A study by de Lima and coworkers (2021)26 showed that both HIIT and MICT can produce improvements in cognitive function and brain-derived neurotrophic factor (BDNF) levels in overweight middle-aged men over a period of eight weeks, three times a week.

**STUDY JUSTIFICATION**

There is still a gap in the modulation of NLRP3 inflammation (and associated interleukins) in obese individuals undergoing HIIT training compared to non-trained individuals. Thus, this study will contribute not only to expanding the understanding of how the inflammasome can be modulated by physical exercise in the context of obesity, but may also assist in the implementation of a non-pharmacological intervention that provides good adherence and brings benefits to an obese population.

**STUDY HYPOTHESIS**

The NLRP3 inflammasome may undergo changes in its expression, which will impact its constituents and associated interleukins (IL-1β, IL-18 and IL-6) after the period of application of the HIIT protocol.

**WORKING PLAN**

1. Inclusion of participants: Obese people who were previously sedentary will take part in the study. They will be divided into two groups: One group will be physically exercised for eight weeks (three times a week) and the other group will not be exercised (control).

2. Evaluation of possible changes through physical exercise: The following analyzes will be performed on obese subjects, both untrained and trained (in this group both before starting the training protocol and after the eight-week training period):

- Analysis of the NLRP3 inflammasome (and associated interleukins - IL-1β, IL-18 and IL-6) by real-time PCR;

- Analysis of the interaction of gene pathways of the NLRP3 inflammasome using the Reactome program.

**GENERAL GOAL**

To investigate possible modulation of the NLRP3 inflammasome (and associated interleukins IL-1β, IL-18 and IL-6) induced by high-intensity interval training (HIIT) in obese individuals compared to untrained individuals.

**SPECIFIC TARGETS**

To evaluate in obese subjects, untrained and trained (in this group both before the start of training and after the eight-week training period):

- Expression of the NLRP3 inflammasome (and associated interleukins - IL-1β, IL-18 and IL-6);

- Interaction of the gene pathways;

- Lipid profile [total cholesterol and fractions (LDL-C and HDL-C) and triglycerides];

- Analysis of body composition by densitometer, by dual-energy X-ray absorptiometry (DEXA).

**METHODS**

**Study design and inclusion and exclusion criteria**

Retrospective study with intervention (trained group) and control groups (untrained group). The chronic effect of eight weeks of high-intensity interval training (HIIT) is investigated.27

The groups consist of obese adults who have not met the World Health Organization (WHO)27 recommendations for physical activity (150 to 300 minutes of moderate/vigorous physical activity (MVPA) per week), of both sexes (18 to 60 years old, with a BMI between 30 and 40 kg/m2).

Cardiovascular risk will be estimated according to the update of the Brazilian Guideline for the Prevention of Dyslipidemia and Atherosclerosis - 201728 and using the ER 2020 calculator for cardiovascular risk stratification, developed by the Atherosclerosis Unit of the Brazilian Society of Cardiology. In addition, a complete physical examination of each participant will be performed, following the standards of the American Heart Association and the Brazilian Society of Cardiology, which is their priority:

- Assessment of vital signs.

- Palpation and auscultation of the pulses.

- Observation of the veins.

- Inspection and palpation of the chest.

- Percussion, palpation and auscultation of the heart.

- Examination of the lungs, including percussion, palpation and auscultation.

- Examination of the abdomen and limbs.

In addition to these data, anthropometric assessment [measurement of body mass, height and body mass index (BMI)] was performed at the same visit. The participants in the trained group are clinically monitored by the medical team during the eight weeks of the training protocol.

People with diabetes, people taking statins, people being treated for obesity or who have undergone bariatric surgery, people who have participated in another regular exercise program in the six months prior to the start of the intervention period (e.g. aerobics and/or strength training). Aerobic and/or strength training); individuals with severe motor disabilities, musculoskeletal injuries, severe respiratory disease, cardiac disease (severe heart failure, cerebrovascular disease, severe cardiopulmonary dysfunction, acute myocardial infarction or early phase of rehabilitation, untreated malignant arrhythmias, untreated severe hypertension, severe pulmonary hypertension), stroke or neurological disease, symptomatic cardiac malformations (such as septal defects, patent ductus arteriosus or valvular stenosis) are excluded. Volunteers who participate in less than 80% of the training days or who do not participate in training for 3 consecutive days will be excluded from the study.

Participants are recruited from the PAEC - Community-Based Sports Activities Program database and waiting lists. PAEC is a UNISA project that offers sports activities to improve the quality of life and well-being of the community in the southern region of São Paulo and the UNISA academic community.

Participants read and signed the Free and Informed Consent Form (FICF). The study was approved by the Research Ethics Committee of the Universidade Santo Amaro and registered as a clinical trial (Brazilian Registry of Clinical Trials – ReBEC) prior to its start.

Sample size calculation

When calculating the sample size, an effect size of 0.55, a significance level (α) of 5 %, a confidence interval of 90 % and a study power of 80 % were taken into account, so that the number of 84 participants was reached (G*Power, version 3.1.9.4).29

The randomization of the groups is carried out using the jerrydallal platform, which can be accessed via the following link <http://www.jerrydallal.com/random/randomize.htm>.

**Assessment of baseline levels of daily physical activity and cardiorespiratory functioning**

The International Physical Activity Questionnaire (IPAQ),30 a valid and reproducible instrument for measuring the level of physical activity in the Brazilian population, is used to determine the level of daily physical activity of all subjects participating in the study. The IPAQ consists of eight questions on walking time, moderate physical activity and vigorous physical activity (sum of both = MVPA), which were carried out in the last seven days.

After using the IPAQ, all subjects undergo the 6-minute walk test, which measures distance walked, heart rate, blood pressure, oxygen saturation with an oximeter and subjective perception of exertion (Borg scale). It is worth mentioning that the 6-minute walk test is performed before starting the exercise protocol and after eight weeks after completing the protocol for the trained group to assess whether the exercise of the physical exercise program has led to an improvement in the functional physical fitness of the subjects.

**Protocol for physical activity**

For eight weeks, the physical exercise program is performed three times a week on a cycle ergometer (Vertical Ergometric Bike V3, Movement, Brazil), following a recommended protocol that involves a gradual increase in the stimulation time and repetitions of the training used. A five-minute warm-up phase is performed at the beginning and end of each training session, after which the subjects return to rest. As for the training itself, to adapt to the protocol, during the first week the subject is subjected to 4 stimuli of 1 minute at high intensity (80 to 100% maximum heart rate - HRmax), followed by 3 minutes of recovery (50 to 70% HRmax). In the second week, the subject is exposed to 6 stimuli of 1 minute at high intensity (80 to 100% maximum heart rate - HRmax), followed by 3 minutes of recovery (50 to 70% HRmax). In the third week, the subject is exposed to 6 stimuli of 1 minute and thirty seconds at high intensity (80 to 100% maximum heart rate - HRmax), followed by 3 minutes of recovery (50 to 70% HRmax). From the fourth week and until the end of the training period, the subject is exposed to 6 stimuli of 2 minutes at high intensity (80 to 100% maximum heart rate - HRmax), followed by 3 minutes of recovery (50 to 70% HRmax).

It is worth noting that the most direct and recognized method for determining HRmax is related to the ergospirometric test. However, for individuals with risk factors such as obesity, there is an obvious concern that they perform a maximal effort test, i.e. that they reach 100% of their HRmax. Therefore, for this population, an alternative is to perform a test that is usually terminated when the individual reaches a predetermined percentage of HRmax, usually around 85% of the predicted HRmax.31 Although a less worrisome test may be used, the determination of HRmax is performed in accordance with the guidelines for monitoring high-intensity interval training in clinical populations to avoid complications in the study.32 Therefore, the intensity of the stimulus will be monitored using a heart rate monitor with Bluetooth transmission (model H7, Polar, Finland), which will transmit the data to the Polar Team program (Polar Team, Finland) installed on a tablet or smartphone or recorded on the Polar watch, based on the predicted HRmax accompanied by the subjective perception of effort (Borg scale),33,34 allowing confirmation that the target physical zone recommended for each subject was reached in each training session.

Specifically, the determination of HRmax follows the prediction proposed by Barnes et al. (2013)35 for people not taking beta-blockers and the prediction proposed by Brawner et al. (2004)36 for people taking beta-blockers, as shown below:

- People not taking beta-blockers: 221 - (0.64 x age)

- People taking beta-blockers: 164 - (0.7 x age)

The Borg Scale (or Perceived Exertion Scale) consists of a numerical scale of 6 to 20 points on which the person performing the exercise session verbally reports their perception of exertion. Subjects should keep in mind that 6 points means “no effort” and 20 points means “maximum effort”. It is expected that the effort during the physical training session will be between 17-18 points. The scale is used after each high-intensity stimulus and at the end of the recovery phase. If the perception during the high-intensity stimulus is less than 17-18 points, the workload should be increased by 1 to 3% at the beginning of the next stimulus.

Finally, all participants are supervised and monitored by the same sports instructor.

**Analysis of body composition**

Body composition will be determined with a densitometer using dual-energy X-ray absorptiometry (DEXA) at the first visit (in both the trained and untrained groups) and after eight weeks of training (in the trained group). The total body image will be recorded using GE Healthcare's Lunar device (iDXA Madison, WI, USA) and the parameters related to the concentration of lean mass and fat of each subject will be evaluated. In order to check whether the training program has led to an improvement in obesity parameters, not only will data on body mass index (BMI) be collected, but the subjects in the trained group will also undergo a new body assessment using DEXA eight weeks after completing the training protocol.

**Blood collection**

Peripheral blood samples are taken in a fasting state before the start of the training protocol (at the beginning, for both the trained and untrained groups) and 48 hours after the last training session (in this case only for the trained group, after eight weeks of training), specifically to investigate the chronic effects of training. Samples will be collected in appropriate tubes to obtain serum and/or plasma aliquots (at least 500 μl) from the clotted or non-clotted blood in the collection tube itself and after centrifugation at 2500 rpm for 10 minutes at 4ºC, and then frozen in liquid nitrogen for later analysis of the lipid profile and NLRP3 inflammasome, as described below.

**Laboratory studies**

The lipid profile (total cholesterol and fractions, triglycerides), fasting blood glucose and glycosylated hemoglobin are determined with commercially available kits (Labtest) and measured with a spectrophotometer.

**Obtaining mononuclear cells from peripheral blood**

Approximately 15 ml of peripheral blood is taken from the study participants on an empty stomach in tubes containing EDTA (to prevent clotting) at the first examination (for both groups) and at the end of the eight-week physical exercise (for the trained group). The samples are mixed with Ficoll-Hypaque (Ficoll Paque Plus, GE Healthcare Bio-Sciences AB, Uppsala, Sweden) and centrifuged at 800 g, 22°C for 20 minutes, separating the mononuclear cells by a concentration gradient. The recovered cells are washed in isotonic solution - PBS. Cell viability and cell count are determined in a Neubauer chamber (hemocytometer) after staining 10 uL of cells with 90 uL of 60% trypan blue (Sigma-Aldrich, MO, USA) for five minutes. The samples are then centrifuged and 1 mL of freezing solution (10% DMSO + 90% fetal bovine serum) is added and the cells are stored in liquid nitrogen.Análise do inflamassoma NLRP3 (e interleucinas associadas: IL-1β, IL-18 e IL-6)

***RNA isolation***

To study the NLRP3 inflammasome (and associated interleukins - IL-1β, IL-18 and IL-6), mononuclear cells are kept at room temperature for 10 minutes for RNA isolation. 200 uL of chloroform is added to each sample, homogenized in a vortex for two minutes, kept at room temperature for five minutes and then centrifuged (20 minutes, 13,000 rpm, 4°C). The clear part is transferred to new Eppendorf tubes and 300 uL isopropanol is added. The samples are again homogenized in a vortex for two minutes, kept on ice for 10 minutes and centrifuged (20 minutes, 13,000 rpm, 4°C). The supernatant is removed and the pellet is washed twice with buffer containing 70% isopropanol + 30% DNAase and RNAase-free water (Invitrogen). The samples are centrifuged again (20 minutes, 13000 rpm, 4ᴼC), the supernatant is removed and the tubes containing the samples are kept open for 30 minutes (to evaporate the isopropanol). At the end, the pellet is resuspended in 20 uL of DNAse and RNAse-free water, the samples are heated to 650C and stored at -800C.

***Reverse transcription***

The RNA concentration in each sample is determined using the Nanodrop 2000 device (Applied Biosystems). The cDNA is extracted from 1 ug of each RNA sample using the Reverse Transcription System Kit (Promega).

***Real-Time PCR***

Experiments are performed in triplicate, at the first visit (for both groups) and after eight weeks of exercise (for the trained group). Each sample contains SYBR Master Green Mix (Applied Biosystems), DNAse and RNAse free water, 1 uL of cDNA and 0.2 uL of each primer (total volume of each sample: 20 uL). Table 1 shows the primers used.

The reactions are analyzed using the 7500 Fast Real Time PCR system and the Fast System SDS software (Applied Biosystems).

**Table 1.** Genes and respectives primers

| **Gene** | ***5’-3’ Forward primer*** | ***5’-3’ Reverse primer*** |
| --- | --- | --- |
| NLRP3 | ATGCCAGGAAGACAGCATTG | TCATCGAAGCCGTCCATGAG |
| ASC | AACCCAAGCAAGATGCGGAAG | TTAGGGCCTGGAGGAGCAAG |
| CASP1 | GGACAAACCGAAGGTGATCATC | TAGCATCATCCTCAAACTCTTCTGTAGT |
| IL-1β | GTCACAAGAAACCATGGCACAT | GCCCATCAGAGGCAAGGA |
| IL-18 | GACAGCCTGTGTTCGAGGAT | TGGATCCATTTCCTCAAAGG |
| IL-6 | CTGCAAGAGACTTCCATCCAGTT | AGGGAAGGCCGTGGTTGT |
| 18 S* | GTAACCCGTTGAACCCCATT | CCATCCAATCGGTAGTAGCG |

* *housekeeping gene.*^37,38^

**Analyzing the interaction between genes and pathways**

In order to better understand how the parameters to be evaluated in this study (NLRP3 inflammasome and associated interleukins - IL-1β, IL-18 and IL-6) may be related to specific gene pathways, a gene interaction analysis will be performed using the program Reactome (reactome.org). With this program it will be possible to analyze the expression profile of these parameters and their relationship to canonical pathways as well as the interaction between different pathways. It is expected that at the end of the analyzes, the most important signaling pathways that may be altered in trained and untrained individuals can be identified.

In this way, the gene expression panel and other parameters that are evaluated here are loaded into the program in the form of a spreadsheet containing this information as well as the results of the statistical analyzes. Once this spreadsheet has been loaded into IPA, the core analysis is performed. The most important altered signaling pathways, possible relationships to diseases and the pathways of gene interactions of these signaling pathways are determined.

Finally, the pathways will be expressed in images with connected boxes and colors that differentiate the genes with greater and lesser expression, as well as a summary of the functions of each of the altered pathways and the functions of each gene within the pathways. In addition, the differentiation in expression levels can also be expressed in heatmap format.

**Statistical Analysis**

For the analysis of descriptive measures and statistical tests, the SPSS version 23.0 program will be used, using parametric and nonparametric tests according to the nature of the variables.

To test the normality of the variables, the Kolmogorov-Smirnov test will be used. Descriptive data will be presented as percentage, mean ± standard deviation, or median and interquartile range, when appropriate. Comparisons between groups (intervention and control) will be made using the unpaired t-test or Mann-Whitney test, and the paired t-test and Wilcoxon test will be used for comparisons between visits in the trained group (initial visit, before the start of the training protocol and at the end of the eight weeks of training). Significance level will be set at p < 0.05.

**References**

1. Lin X, et al. Obesity: Epidemiology, pathophysiology, and therapeutics. *Front Endocrinol* 2021;12:706978.

2. Singh GM, et al. The age-specific quantitative effects of metabolic risk factors on cardiovascular diseases and diabetes: a pooled analysis. *PLoS One* 2013;8:e65174.

3. Powel-Willey TM, et al. Obesity and cardiovascular disease: A scientific statement from the American Heart Association. Circulation 2021;143(21):e984-e1010.

4. Jensen MD, et al. 2013 AHA/ACC/TOS guideline for the management of overweight and obesity in adults: a report of the American College of Cardiology/American Heart Association Task Force on Practice Guidelines and The Obesity Society. Circulation 2014;129(suppl 2):S102– S138.

5. Guo H, et al. Inflammasomes: mechanism of action, role in disease, and therapeutics. Nat Med 2015;21:677–687.

6. Duewell P, et al. NLRP3 inflammasomes are required for atherogenesis and activated by cholesterol crystals. Nature 2010;464:1357–1361.

7. Swanson KV, et al. The NLRP3 inflammassome: molecular activation and regulation to therapeutics. Nat Rev 2019;19:477-489.

8. Sokolova M, et al. NLRP3 inflammasome: A novel player in metabolically induced inflammation – potential influence in myocardium. J Cardiovasc Pharmacol 2019;74:276-284.

9. Sehgal A, et al. Targeting NLRP3 inflammasome as a chief investigator of obesity, contributing to local adipose tissue inflammation and insulin resistance. Environ Sci Pollut Res Int 2021;28:43102-43113.

10. Stienstra R, et al. Inflammasome is a central player in the induction of obesity and insulin resistance. Proc Natl Acad Sci U S A. 2011;108:15324-15329.

11. Unamuno X, et al. NLRP3 inflammasome blockade reduces adipose tissue inflammation and extracellular matrix remodeling. Cell Mol Immunol 2021;18(4):1045-1057.

12. Aday AW, et al. Antiinflammatory therapy in clinical care: The CANTOS trial and beyond. Front Cardiovasc Med 2018;5:62.

13. Ridker PM, et al. Antiinflammatory therapy with Canakinumab for atherosclerosis disease. New Engl J Med 2017;377:1119-1131.

14. Rothman AMk, et al. Effects of interleukin-1β inhibition on blood pressure, incidente hypertension, and residual inflammatory risk: A secondary analysis of CANTOS. Hypertension 2020;75:477-82.

15. Ridker PM, et al. Modulation of the interleukin-6 signalling pathway and incidence rates of atherosclerotic events and all-cause mortality: analyses from the Canakinumab anti-inflammatory thrombosis outcomes study (CANTOS). Eur Heart J 2018;39:3499-3507.

16. Broch K, et al. Randomized trial of interleukin-6 receptor inhibition in patients with acute ST-segment elevation myocardial infarction. J Am Coll Cardiol 2021;77(15):1845-1855.

17. Akra S, et al. The NLRP3 inflammasome activation in subcutaneous, epicardial and pericardial adipose tissue in patients with coronary heart disease undergoing coronary by-pass surgery. Atheroscler Plus 2022;48:47-54.

18. Mejías-Peña Y, et al. Impact of resistance training on the autophagy-inflammation-apoptosis crosstalk in elderly subjects. Aging 2017;9:408-18.

19. Zhang T, et al. Exercise training-attenuated insulin resistance and liver injury in elderly pre-diabetic patients correlates with NLRP3 inflammasome. Front Immunol 2023;14:1082050.

20. Abkenar IK, et al. The effects of acute and chronic aerobic activity on the signaling pathway of the inflammasome NLRP3 complex in young men. Medicina 2019;55:105.

21. Armannia F, et al. Effects of High-Intensity Interval vs. Moderate-Intensity Continuous Training on body composition and gene expression of ACE2, NLRP3, and FNDC5 in obese adults: A randomized controlled trial. Med J Islam Repub Iran 2022;36:161.

22. Sabag A, et al. The effect of High-Intensity Interval Training vs Moderate-Intensity Continuous Training on liver fat: A systematic review and Meta-Analysis. J Clin Endocrinol Metab 2022;107(3):862-881.

23. Weston KS, et al. High-intensity interval training in patients with lifestyle-induced cardiometabolic disease: a systematic review and meta-analysis. Br J Sports Med 2013;48:1227-34.

24. Batacan RB, et al. Effects of high-intensity interval training on cardiometabolic health: a systematic review and meta-analysis of intervention studies. Br J Sports Med 2017;51(6):494-503.

25. Maillard F, et al. Effect of high-intensity interval training on total, abdominal and visceral fat mass: a meta-analysis. Sports Medicine 2018;48:269-88.

26. de Lima NS, et al. Moderate-Intensity Continuous Training and High-Intensity Interval Training improve cognition, and BDNF levels of middle-aged overweight men. Metab Brain Dis 2022;37:463-471.

27. Bull FC, et al. World Health Organization 2020 guidelines on physical activity and sedentary behaviour. Br J Sports Med 2020;54:1451-62.

28. Faludi AA, et al. Atualização da Diretriz Brasileira de Dislipidemias e Prevenção da Aterosclerose - 2017. Arq Bras Cardiol 2017;109:1-92.

29. Faul F, et al. G*Power 3: a flexible statistical power analysis program for the social, behavioral, and biomedical sciences. Behav Res Methods. 2007;39:175-91.

30. Craig CL, et al. International physical activity questionnaire: 12-country reliability and validity. Med Sci Sports Exerc 2003;35:1381-95.

31. Balady GJ, et al. Clinician’s guide to cardiopulmonary exercise testing in adults: a scientific statement from the American Heart Association. Circulation 2010;122(2):191-225.

32. Taylor JR, et al. Guidelines for the delivery and monitoring of high intensity interval training in clinical populations. Prog. Cardiovasc Dis 2019;62(2):140-6.

33. Borg G. Borg's perceived exertion and pain scales. Human kinetics, 1998.

34. Borg G. Perceived exertion as an indicator of somatic stress.Scand J Rehab Med, 1970.

35. Barnes BM, et al. Age‐predicted maximal heart rate in healthy subjects: The HUNT F itness S tudy. Scand J Med Sci Sports 2013;23(6):697-704.

36. Brawner CA, et al. Predicting maximum heart rate among patients with coronary heart disease receiving β-adrenergic blockade therapy.Am Heart J 2004;148(5):910-14.

37. Murphy AJ, et al. IL-18 production from the NLRP1 inflammasome prevents obesity and metabolic syndrome. Cell Metab 2016;23:155–64.

38. Kim Y, et al. Suppression of NLRP3 inflammasome by γ-tocotrienol ameliorates type 2 diabetes. J Lip Res 2016;57:66-76.

39. Yang XT, et al. Differential cytokine expression in gastric tissues highlights *helicobacter pylori’s* role in gastritis. Sci Rep 2024;14:7683.
