# Supplementary material for: The effects of high-intensity interval training on NLRP3 inflammasome and monocyte chemokine receptors in individuals with obesity
Source: PLoS One. 2026 Feb 23;21(2):e0343214. doi: 10.1371/journal.pone.0343214 (PMC12928487; doi:10.1371/journal.pone.0343214)
Supplement: S2 Table — (DOCX) [file pone.0343214.s003.docx]

**Supplementary Table 2.** Genes and respective *primers* of the study

| **Gene** | ***5’-3’ Forward primer*** | ***5’-3’ Reverse primer*** |
| --- | --- | --- |
| NLRP3 | ATGCCAGGAAGACAGCATTG | TCATCGAAGCCGTCCATGAG |
| ASC | AACCCAAGCAAGATGCGGAAG | TTAGGGCCTGGAGGAGCAAG |
| CASP1 | GGACAAACCGAAGGTGATCATC | TAGCATCATCCTCAAACTCTTCTGTAGT |
| CCR2 | ATGCTGTCCACATCTCGTTCTCG | TTATAAACCAGCCGAGACTTCCTGC |
| CCR5 | CACCTGCAGCTCTCATTTTCC | TTGTAGGGAGCCCAGAAGAG |
| CX3CR1 | CCCTGAATCAGTGACAGAAAACT | ACGGAGTAGAATATGGACAGGAA |
| 18 S* | GTAACCCGTTGAACCCCATT | CCATCCAATCGGTAGTAGCG |

* *housekeeping gene*
