# Supplementary figures and images for: The effects of high-intensity interval training on NLRP3 inflammasome and monocyte chemokine receptors in individuals with obesity
Source: PLoS One. 2026 Feb 23;21(2):e0343214. doi: 10.1371/journal.pone.0343214 (PMC12928487; doi:10.1371/journal.pone.0343214)

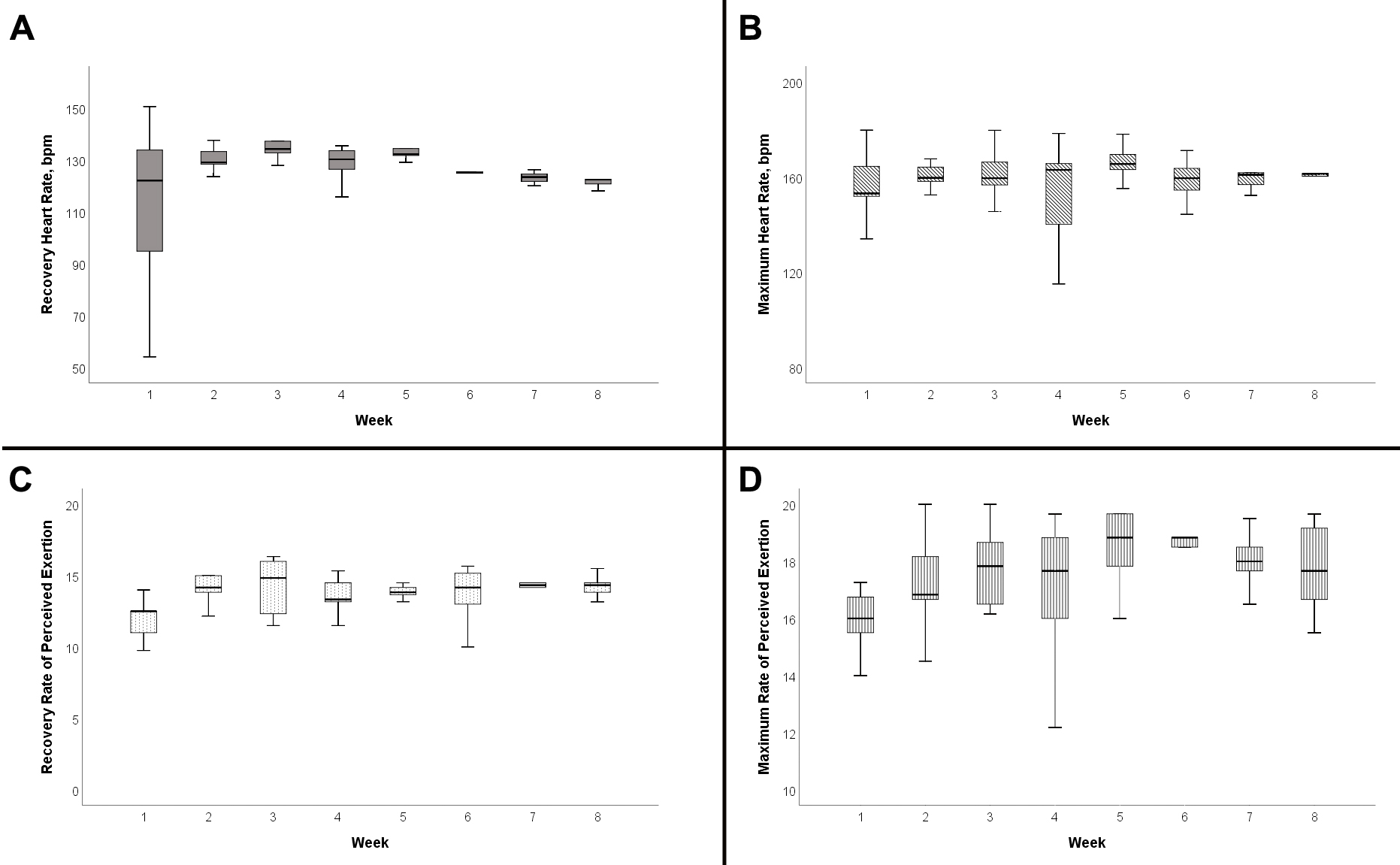

Supplement: S1 Fig — A) Recovery Heart Rate, B) Maximum Heart Rate, C) Recovery Rate of Perceived Exertion; D) Maximum Rate of Perceived Exertion. (JPG) [file pone.0343214.s005.jpg]

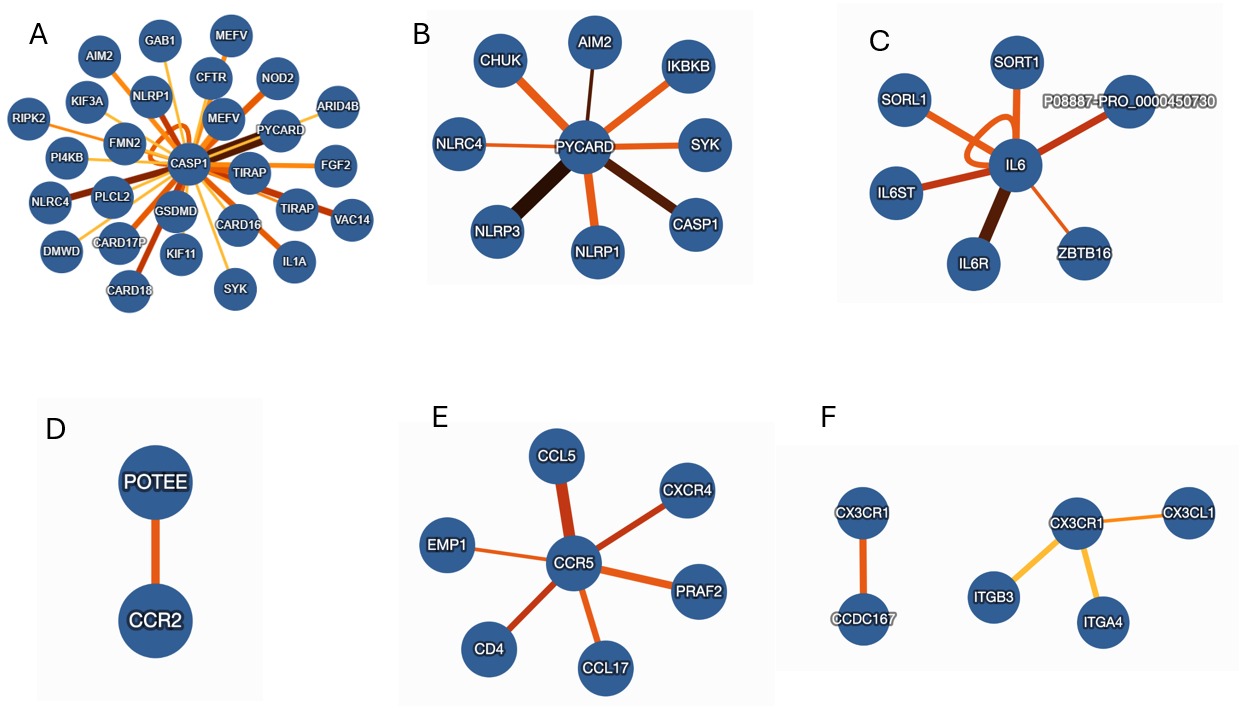

Supplement: S2 Fig — A) CASP-1; B) PYCARD; C) IL-6; D) CCR2; E) CCR5; F) CX3CR1. (JPEG) [file pone.0343214.s006.jpeg]
